# Naturalist's Monthly Report

**Published:** 1812-02

**Authors:** 


### NATURALIST'S MONTHLY REPORT.

### DECEMBER.

#### DEAD-WINTER MONTH.

The field has lost its verdure: all the pride
Of the sweet garden fades. Where now the rose,
The pink, the stock, the lupin, or the pea?

FROM the 1st to the 4th of the month the wind was westerly; on the 5th north-north-east; from the 5th to the 9th westerly or south-west; on the 10th variable; on the 11th and 12th easterly; from the 13th to the 20th westerly or south-west; from the 21st to the 24th north-west; on the 25th and 26th easterly; on the 27th variable; on the 28th, 29th, and 30th, north-east; and on the 31st variable.

The winds have been more than usually boisterous. On the 1st, 2d, 3d, 7th, 16th, 20th, 27th, and 28th, we had strong gales; and on the 4th, 8th, 9th, squally weather.

Rain, more or less, fell on the 1st, 3d, 4th, 6th, 8th, 9th, 10th, 12th, 13th, 14th, 15th, 16th, 17th, 18th, 19th, 20th, and 28th.

There was a sharp frost on the 5th, 21st, 22d, 26th, 27th, 28th, 29th, and 30th; and some snow in the evening of the 27th, but this was all dissolved in the course of the following day.

December

December 1st. House-flies still continue occasionally to buzz about in warm rooms; but they appear very weak and languid. A large blue-bottle fly (*musca vomitoria* or *carnaria*) came out of its hiding-place and flew about the candles.

December 6th. Several martins (*hirundo urbica*) have been observed in flight in the course of the last six or seven days. They were seen about the middle of each day, flying over the houses, and about the church, as in the middle of summer. They appear to have finally retired either on the 5th or the 6th.

December 8th. A few herrings continue to be left on this coast, but the principal shoals have taken their departure, either into the deep water or distant seas.

December 11th. The redbreast sings. Kingfishers are seen about the rivers.

December 12th. The skylark sings. A great number of bulfinches have, within the last few days, migrated into this neighbourhood.

December 18th. The weeping-willow, elder, and laburnum, trees, are putting forth their leaves. Indeed the mildness of the weather has of late been such, that vegetation in general is nearly in a state as forward as it usually is about the latter end of March or beginning of April.

December 21st. Gnats fly about in the night. Moths of various kinds appear. Gooseberry-trees are in bloom; and upon some of them the fruit is even set. Several species of garden plants are yet in flower. *Daphne collina* is in bloom in the open ground.

December 24th. I have just been informed of a singular instance of instinct in the common sparrow. A few days ago a sparrow was remarked to fly several times, with food in her mouth, into a hole in an old wall. The curiosity of the person observing it was excited to ascertain the cause; and, ascending to the place with a ladder, he found there a full grown bird of the summer's brood, which had been accidentally entangled by one leg, in such manner as not to be able to escape. Thus fettered, the old birds had not forsaken their unfortunate offspring, but had continued to feed and support it in its confinement, even for so many months after all the other individuals of the same brood had taken their flight.

December 27th. The ice is sufficiently thick to allow of persons sliding and skaiting upon the ponds and slowly running streams. Although it is stated that, at the distance of a few miles from the sea-coast there has been a great fall of snow, we have had none in this neighbourhood till to-day. By the late frosty weather the too great progress of vegetation has been completely checked.

December 29th. The radical leaves of several of the early flowering plants are beginning to appear out of the ground. The woodbines have already put forth their first leaves.

December 31st. I have not been informed that any species of wild geese have yet been seen in this immediate neighbourhood during the present month. Wild ducks, wigeons, and teale, are also said to have been extremely scarce.

*Hampshire.*

